# Long-Term Field Study Reveals Subtle Effects of the Invasive Alga *Sargassum muticum* upon the Epibiota of *Zostera marina*

**DOI:** 10.1371/journal.pone.0137861

**Published:** 2015-09-14

**Authors:** Stacey DeAmicis, Andrew Foggo

**Affiliations:** 1 Marine Biology & Ecology Research Centre, School of Marine Science & Engineering, Plymouth University, 620 Davy Building, Drake Circus, Plymouth, United Kingdom; 2 Marine Biology & Ecology Research Centre, School of Marine Science & Engineering, Plymouth University, 617 Davy Building, Drake Circus, Plymouth, United Kingdom; Texas A&M University at Galveston, UNITED STATES

## Abstract

Invasive species can alter coastal ecosystems both directly, e.g. through competition for substratum and nutrients, and indirectly. Indirect effects may be mediated by creation of dissimilar or inimical habitats, changes in predator and/or prey assemblages, alterations in associated biota, and perturbations of water movement and thermal regimes. Previous studies have shown that invasive algae can modify native habitat architecture, disrupt intricately linked food webs and alter epibiotic assemblages. In the UK, the seagrass *Zostera marina* supports a diverse epibiotic assemblage, influencing key factors such as sediment dynamics, depositional regime and trophic linkages. Increasing encroachment of the invasive alga *Sargassum muticum* into seagrass meadows changes the physical and chemical characteristics of the local environment and creates the potential for changes in the epibionts associated with the seagrass blades, threatening the integrity of the seagrass ecosystem. We investigated the effects of *S*. *muticum* invasion upon the epibiota of *Z*. *marina* in a drowned river valley in SW England seasonally from spring to autumn over four years in an *in-situ* manipulative experiment, comparing permanent quadrats with and without artificially introduced *S*. *muticum*. Epibiota were weighed, identified to the most detailed operational taxonomic unit (OTU) possible, and unitary organisms were enumerated. Multivariate PERMANOVA+ analysis revealed significant differences in epibiont assemblages between *Sargassum* treatments. Linear mixed effects models indicated that differences in epibiota assemblage composition were not reflected as significant differences in mean biomass per sample, or number of epibiont OTUs per sample. We conclude that *S*. *muticum* invasion into *Z*. *marina* meadows may significantly alter the species composition and abundance distribution of epibiotic assemblages found on the blades of the seagrass. Thus *S*. *muticum* invasion could have more wide-reaching effects on processes within coastal ecosystems than predicted purely by direct effects.

## Introduction

Invasive species and their effects on ‘natural’ terrestrial environments have been widely studied with results indicating that terrestrial habitat-forming invasive species have large effects on native biota [1,2]. In comparison, our understanding of the impacts of invasive plants and algae in coastal marine ecosystems is less well developed, with fewer studies concerning how and to what extent these invaders modify the composition and function of native communities [3–5]. As the number of reported marine invasions increases due to the continuing rise in global transportation [6–8] it is unlikely that any coastal ecosystem will escape the effects of invasive species.

Seagrass meadows provide a heterogeneous, complex habitat matrix and play host to an extensive and diverse range of biota found on and amongst their leaves including cyanobacteria [9], diatoms [9,10], epiphytic algae [11], and sessile as well as mobile epifauna [12–14]. They also provide sheltered habitat for larval fish and oviposition substrata for many different organisms such as molluscs and fish [15–17]. Part of the importance of seagrass meadows is derived from the architectural complexity they add to soft substratum habitats [18]. Generally, the greater the complexity, the higher the species abundance and diversity [19]; epibiotic species richness and abundance both add to this complexity and can vary markedly between locations and from season to season [20,21]. Although seagrasses provide a complex habitat, they are generally a poor food source for grazers [16,22]. Their epibionts, by contrast, contribute significantly to the flux of energy and nutrients to higher trophic levels [22–25] as well as having significant effects upon sedimentation processes and flow rates [26] by increasing the total surface area and complexity of the seagrass ‘blades’.

Marine macrophytes such as *Sargassum muticum* (Yendo) Fensholt and other phaeophytes which may occur in close proximity to seagrasses can also host a diverse assemblage of epibionts [3,21,23] which themselves may impact upon seagrass ecosystems, for example in regions where a macrophyte is non-native it may support additional non-native species [27–29]. Ecological problems arise when non-native algal species establish themselves in ‘new’ habitats, altering the native habitat architecture and potentially disrupting intricately linked food webs [30]. Previous research has shown that invasive macroalgae have the potential to change epifaunal communities [3,14,28,31], and thereby alter entire ecosystems [14,31]. As invasive macroalgae such as *S*. *muticum* become more cosmopolitan in distribution, even invading soft substrate habitats like seagrass beds [32], the need to elucidate the impacts of such invasions upon seagrass systems becomes more pressing.

With its high fecundity and rapid annual growth to dimensions exceeding those normally documented in its native range (authors’ own observations), *S*. *muticum* has the potential to adversely affect *Zostera marina* L. and its associated epibiotic assemblages [33] in the NE Atlantic through processes such as shading [34] and allelopathy [35]. The Salcombe-Kingsbridge system has been experiencing a dramatic increase in *S*. *muticum* density in recent years [32], with densities during the study peaking at 4 thalli / m^2^. This spread has been achieved largely through vegetative reproduction (fragmentation of mature specimens) and settlement of these fragments upon small stones and gastropod shells, followed by peripatetic dispersal during which these small anchors become entrapped amongst the seagrass root-rhizome matrix [32] and buried allowing proliferation of the alga. With the introduction and establishment of this dominant macrophytic alga, the magnitude of the effects from such an invasion upon the epibiota of *Z*. *marina* will depend on their ability to withstand: i) the increased shading provided by the greater architectural complexity and buoyancy of *S*. *muticum* [36–39]; ii) the novel biochemistry the alga adds into the local environment [40,41]; iii) new biota capable of using the alga as a substrate or altered assemblages of seagrass epibiota facilitated by the invasive alga. The aim of this four year field study was therefore to investigate how the presence of *S*. *muticum* affects the associated epibiota of *Z*. *marina*. We addressed the following specific questions: i) do epibiotic assemblages and number of types of epibionts differ in the presence/absence of *S*. *muticum*? ii) if species assemblages are different, how do they differ? iii) does the presence of *S*. *muticum* affect the epibiota biomass and number of epibiont types? and iv), what inter-seasonal and inter-annual variability occurs in epiphyte assemblage composition and biomass, and are these different in the presence/absence of *S*. *muticum*?

## Methods

The study was conducted around Woodville Rocks in the Salcombe-Kingsbridge drowned river valley, SW England (50°13′53″N, 03°46′18″W). Salcombe Harbour Authority kindly granted permission to work in the shallow subtidal region of Salcombe-Kingsbridge system. Permission to sample the protected *Zostera marina* was granted by Natural England which designates the site as being of special scientific interest (SSSI) under the Wildlife and Countryside Act 1981.

Twenty randomly located 1 m^2^ permanent quadrats were established in the shallow subtidal (0.5 m below chart datum) in April 2007 and were randomly allocated to two separate treatment groups. All quadrats and the region around them were cleared of naturally occurring *S*. *muticum*, this clearance was repeated at each sampling event to eliminate intrusive influences of naturally occurring *S*. *muticum*. Two intact *S*. *muticum* thalli collected locally were secured at the holdfast onto plastic grids (20 x 20 cm with 5 cm mesh size) and two such grids were pegged into each of 10 of the permanent quadrats to mimic the highest observed local S. muticum density, 4 thalli m^-2^ (*Sargassum* treatment), taking care to feed the seagrass shoots through the mesh also. Two bare plastic grids were anchored into the remaining 10 permanent quadrats in a similar fashion to create control replicates (Control treatment). Seagrass samples were subsequently collected within these quadrats as dictated by tidal windows in three seasons (spring: March–May, summer: June–August and autumn: September–October) during which growing or mature *S*. *muticum* thalli were present in the quadrats over four years (2007–2010). On each sampling occasion all blades from three haphazardly selected *Z*. *marina* shoots within each experimental quadrat were cut directly above the basal meristem (regrowth of cut leaves occurred rapidly ensuring that density changes did not result from sampling) and preserved in 80% alcohol for epibiota analysis. Morphometric data (length, width, area and the number of blades per shoot) were recorded from all blades in each sample prior to preservation. A total of 1135 samples across 18 sampling dates were taken.

### Epibiota

In the laboratory the preservant from each sample was filtered through a small, labelled, pre-weighed dry filter paper which was retained for microscopic analysis and, after inspection and identification, estimation of biomass.

Each blade from each sample was analysed individually in 8 cm sections following Jacobs et al. [42], up to a total of 40 cm per blade. Jacobs et al. [42] demonstrated that epibiotic assemblages can differ between base, middle and tip regions of seagrass blades, therefore blades were analysed according to position along the length: base (basal 8 cm starting at the excision), tip (top 8 cm) and middle region (up to three 8 cm sections). If blades were small (< 24 cm), they were examined in their entirety. A combination of a compound Olympus BHB and Meiji Techno dissecting microscopes were used to identify the epibiota from one side of each blade. All epiphytic algae were classed as either present or absent and if possible, were identified to genus level or to operational taxonomic units (OTUs), otherwise they were classed into six functional groups: filamentous, corticated filamentous, foliose, corticated foliose, saccate and coralline, after Steneck and Dethier [43] and Saunders et al. [44]. Epifauna were identified to species if possible, or to OTU; non-colonial animals were enumerated and colonial animals were classed as present or absent.

After organisms were identified, both sides of each blade were gently scraped using a razor blade to remove epibiota from the blade surface onto a pre-weighed, labelled 9 cm paper filter [19,45,46]. Once the entire sample was processed, both large and small filters for each sample were placed in a Gallenkamp IH-150 drying oven at 50°C for 24 hours. Once dry, the two filters for each sample were weighed, summed, and used to calculate epibiota biomass.

### Data Analysis

#### Epibiota assemblage

Combining data described using different measurement scales creates problems for all statistical analyses; there are few real solutions to the conundrum of testing hypotheses about biota where use of different scales is unavoidable (combining colonial and unitary organisms for example), therefore multivariate statistics including a weighting for abundances were employed. We analysed our complete dataset using a log(X+1) transformation to reduce the influence of abundance [47] prior to analysis in PRIMER v6.1.13 with PERMANOVA+ v1.0.3 (PRIMER-E Ltd, Plymouth, UK). This transformation minimizes problems arising from data measured on different scales (K. R. Clarke, personal communication, 2011). Total examined blade area was used as a covariate to account for the effect of sample size.

A Bray-Curtis similarity resemblance matrix was constructed using transformed data prior to analysis using a four-factor PERMANCOVA+ with type I Sums of Squares, unrestricted permutations of raw data and 9999 permutations [48]. A mixed effects model was applied with Blade Area as a covariate entering the model first, ‘Treatment’ with two levels and ‘Year’ with four levels defined as fixed factors and ‘Season’ with three levels nested within ‘Year’ (collinearity resulted from 2010 data lacking an autumn sample) and ‘Quadrat’ with 20 levels nested within ‘treatment’ as random factors. We also tested for covariate x factor interactions; these were all non-significant. Model simplification (following [48]; threshold for term removal *p* > 0.4) then removed the highly non-significant effect of Year and its interactions, resulting in a final model with Area as a covariate, Treatment and Season as fixed main effects, Quadrat as a random factor nested within Treatment, and the interactions between the fixed effects and between Quadrat within Treatment and Season. All PERMANCOVA main effects were tested for homogeneity of dispersions using PERMDISP tests. The proportional contributions of different epibiota to the dissimilarity between treatments were investigated using SIMPER [47], with an 80% cut-off.

#### Epibiota biomass

Total biomass data were summed to sample level and a linear mixed model (package lme4) [49] was applied in R [50] using the same four-factor design described above. Biomass data were log (X+1) transformed to reduce structure in residuals and achieve the lowest AIC values of the candidate models (including Poisson fit via glmer). Model simplification based upon *p* values (*p* > 0.4) and AICs (ΔAIC > 2) removed the Year effect and then the fixed effects interaction between Treatment and Season resulting in a final model with blade area as a linear covariate, Treatment and Season as fixed main effects and Quadrat as a random factor nested within Treatment. Significance of fixed effects was determined by Likelihood ratio test using the Drop1 function in lme4.

#### Number of epibiota types

The total tally of different organisms (species/OTUs) encountered in each sample was analysed using the same approach described for biomass; data exploration indicated that a Gaussian rather than Poisson model gave the best fit and least structured residuals, and the model simplification processes resulted in the same final model described for biomass above applied to untransformed data.

## Results

### Epibiota assemblage

A total of 226,798 individuals and occurrences, belonging to 87 taxa / OTUs, were identified on the blades of *Z*. *marina* from 1135 samples. After accounting for the effect of Blade Area, PERMANCOVA analysis revealed a significant effect of Treatment (*p* = 0.043, Table 1). Significant effects of Seasons and significant variation among Quadrats within Treatments were also evident (*p* = 0.001 and *p* = 0.015, respectively), however, differences between Treatments were consistent across seasons, and there were no effects of Year (Table 1). Model simplification yielded enhanced Blade Area and Treatment effects but a lessened effect of the random Quadrat term. PERMDISP tests indicated significant heterogeneity of dispersions in both temporal factors but homogeneous dispersion in the Treatment effect. SIMPER analysis revealed greater average similarities within the Control (45.57%) than the *Sargassum* (43.27%) treatment (Table 2); average dissimilarity between the *Sargassum* and Control treatments was 55.66% (Table 3). Copepods, nematodes and foraminifera were consistently the most abundant groups, regardless of treatment. Of the 20 different taxa or OTUs contributing most significantly to between-treatment dissimilarity, 10 had higher densities in the Control treatment, seven were more abundant in the *Sargassum* treatment and three had approximately equal abundances (Table 3). Very few species/OTUs were exclusively found on blades from one treatment or the other; nine species/OTUs were found only within the *Sargassum* treatment and five species/OTUs were found only in the Control treatment.

**Table 1:** PERMANCOVA+ results for epibiota species data (2007–2010). Type I SS PERMANCOVA+ results for epibiota assemblage data from 2007–2010; n = 564 for the *Sargassum* treatment and n = 571 for the Control treatment over 4 years with 3 seasons per year. *P*_*disp*_ gives *P* values for homogeneity of dispersion (PERMDISP) tests for main effects, *P*_*simp*_ gives the *P* values for a simplified model excluding the non-significant effect of Year and its interactions with other main effects.

Source	df	MS	Pseudo-F	*P* (perm)	*P*_*disp*_	*P*_*simp*_
Blade Area	1	9033	1.436	0.178		**0.004**
Treatment	1	4351	1.713	**0.043**	0.701	**0.003**
Year	3	22148	0.310	0.999	**0.001**	
Season(year)	2	57238	47.103	**0.001**	**0.001**	**0.001** *
Quadrat(Treatment)	21	1670	1.340	**0.015**	0.934	0.232
Treatment * Year	3	942	0.908	0.639		
Treatment * Season(Year)	7	1178	0.971	0.522		0.461*
Year * Quadrat(Treatment)	54	1245	1.066	0.259		
Quadrat(Treatment) * Season(Year)	125	1212	0.840	0.994		0.928*
Residual	912	1443				

Note:

* indicates that the random nested season effect is analysed as a fixed effect in the simplified model. In both models all factor * covariate interactions were non-significant.

**Table 2:** SIMPER results for the epibiota similarity within treatments from 2007–2010 long-term field study. Average epibiota similarity within treatments; n = 564 for the *Sargassum* treatment and n = 571 for the Control treatment.

Control **Avg. Similarity = 45.57%**				
**Species**	**Avg. Abundance**	**Avg. Similarity**	**Contribution %**	**Cumulative %**
Diatoms (unidentified)	1.79	7.96	17.47	17.47
Copepods	2.65	5.67	12.45	29.92
Nematodes	2.08	4.42	9.70	39.62
Non-corticated filament	1.28	3.98	8.73	48.35
Sponge	1.12	3.61	7.92	56.27
*Aora gracilis*	1.36	3.54	7.77	64.04
*Porcellidium viridis*	1.02	2.73	6.00	70.03
Foraminifera 1	1.47	2.38	5.23	75.27
Corticated filament	0.98	2.31	5.07	80.34
***Sargassum* Avg. Similarity = 43.27%**				
**Species**	**Avg. Abundance**	**Avg. Similarity**	**Contribution %**	**Cumulative %**
Diatoms (unidentified)	1.78	8.19	18.92	18.92
Copepods	2.39	4.42	10.21	29.14
Non-corticated filament	1.26	4.04	9.33	38.47
Nematodes	1.96	4.01	9.27	47.74
Sponge	1.12	3.84	8.87	56.61
*Aora gracilis*	1.27	3.09	7.14	63.74
*Porcellidium viridis*	1.03	2.79	6.45	70.19
Corticated filament	0.94	2.04	4.71	74.91
Saccate algae	0.87	2.00	4.63	79.54
Foraminifera 1	1.29	1.77	4.08	83.62

**Table 3:** SIMPER results for average epibiota dissimilarity between *Sargassum* and Control treatments from 2007–2010. Average dissimilarity between the two treatments = 55.66%; n = 564 for the *Sargassum* treatment and n = 571 for the Control treatment.

Species/FTU	Control Avg. Abundance	*Sargassum* Avg. Abundance	Avg. Dissimilarity	Contribution %	Cumulative %
Copepods	2.65	2.39	5.87	10.54	10.54
Nematodes	2.08	1.96	4.68	8.41	18.95
Foraminifera ^#^1	1.47	1.29	3.68	6.62	25.57
*Aora gracilis*	1.36	1.27	3.31	5.95	31.52
Ostracods	0.87	0.79	2.56	4.59	36.11
*Porcellidium viridis*	1.02	1.03	2.46	4.42	40.53
Sponge	1.12	1.12	2.24	4.02	44.55
Non-corticated filament	1.28	1.26	2.21	3.98	48.53
Corticated filament	0.98	0.94	2.18	3.92	52.45
Saccate algae	0.85	0.87	2.12	3.81	56.26
Flocculate bacteria	0.58	0.59	1.93	3.47	59.73
Copepod nauplii	0.62	0.62	1.77	3.19	62.92
*Polysiphonia sp*.	0.55	0.55	1.62	2.90	65.82
Bryozoan colony	0.39	0.44	1.36	2.44	68.26
Diatom (unidentified)	1.79	1.78	1.27	2.27	70.53
Stolon tube-like alga	0.36	0.33	1.25	2.24	72.77
*Ceramium sp*.	0.37	0.38	1.20	2.16	74.93
Multi-cellular blade (foliose)	0.36	0.38	1.17	2.11	77.04
Nereidae	0.27	0.31	1.00	1.79	78.83
*Licmophora sp*.	0.27	0.22	0.96	1.72	80.55

### Epibiota biomass

The linear mixed effects model indicated no significant effects of Treatment upon epibiota biomass (Likelihood Ratio = 3.55, 1 *d*.*f*., *p* = 0.059), but blade area (Likelihood Ratio = 335.80, 1 *d*.*f*., *p* < 0.001) and seasonal (Likelihood Ratio = 129.28, 2 *d*.*f*., *p* < 0.001) effects were strong.

### Number of epibiota types

The linear mixed effects models indicated no significant effects of Treatment upon number of epibiota types (Likelihood Ratio 1.345, 1 *d*.*f*., *p* = 0.246), but blade area (Likelihood Ratio 102.01, 1 *d*.*f*., *p* < 0.001) and seasonal (Likelihood Ratio 65.654, 2 *d*.*f*., *p* < 0.001) effects mirrored those for biomass.

## Discussion

Seagrass beds with high shoot and blade densities as well as dense root-rhizome matrices provide rich habitat with high associated diversity [44,51]. Results from this field study found at least 87 different epibiota species/OTUs, suggesting that *Z*. *marina* may play a significant role in providing habitat or substratum for many different organisms within the system. We found that in the Salcombe-Kingsbridge system the mean total surface area shoot^-1^ m^-2^ for *Z*. *marina* declined over the duration of the long-term field study as a result of a decrease in blade length [52]. With decreasing area available for epibiota to colonise, changes within the epibiotic assemblage of *Z*. *marina* may already be under way. Organisms that rely upon the seagrass may be affected due to loss of substrata for egg deposition [3] and loss of preferred food and habitat [28,53] due to declining *Z*. *marina* densities and decreasing blade lengths coupled with increasing numbers of *S*. *muticum* thalli within the estuary [52].

Our results are consistent with previous findings describing impacts of invasive macrophytic seaweeds upon associated biota in seagrass beds [54] and areas of native seaweeds [28,29,53]. The first primary mechanism we postulate that may underlie these impacts is change in habitat architecture (i.e. complexity / heterogeneity and availability) [19,27,29,55]. The habitat diversity hypothesis suggests that the species-area relationship changes with the sampling area size [56]; species diversity then increases with increasing spatial area and is controlled by the availability of differing habitats for colonisation by different species [56,57]. Habitat architecture drives faunal composition at the micro-scale whereas habitat size drives faunal abundances [58] as well as influencing composition via the colonisation ‘lottery’. Previous research has reported epibiotic species richness and composition change when habitat complexity increases [19,21,27,55]. Although the study area (i.e., the permanent quadrats) did not increase in size, per se, the potential colonisable space available within the quadrats was likely increased in the *Sargassum* treatment due to the presence of added *S*. *muticum* (note however that mean area for colonisation available on *Z*. *marina* only remained comparable across treatments—159 cm^-2^ quadrat^-1^ in the Control treatment vs 158 cm^-2^ quadrat^-1^ in *Sargassum* treatment) [52]. Buschbaum et al. [53] and Gestoso et al. [27] found that *S*. *muticum* increased habitat heterogeneity and substratum availability, and thus supported greater epibiota species richness than native algae. Schmidt and Scheibling [59] also found higher epibiont diversity on the invasive *Codium fragile subsp*. *tomentosoides* (van Goor) P. C. Silva compared to the native kelps (*Laminaria sp*.) in Canadian waters, whereas Lutz et al. [31] found that species richness did not differ between the native and invasive species of macroalgae studied in Australia. Norton and Benson [54] found similarities between the fauna that inhabited *Z*. *marina* beds and the fauna found on *S*. *muticum* and proposed that the seagrass beds ‘restocked’ the stands of *S*. *muticum* around Friday Harbor, WA. It is clear therefore that although effects are likely to be highly variable and context-specific, the majority of studies demonstrate that invasive macrophytes generally do affect native epibiotic assemblages.

Results from our long-term study indicated that the epibiotic assemblage in quadrats colonised by *S*. *muticum* differed from controls largely in distribution of abundances as well as species/OTU composition, with a small number of epibiota species/OTUs (9 total: see Table 3) accounting for over 50% of the differences between the two treatments. This result corresponds with previous findings by Gestoso et al. [27,28] who reported that the abundance of epibiota species, not the species composition of epibiota differed between *S*. *muticum* and two native macrophytic algae in northern Spain. Interestingly, despite the differences in the taxa present in our study, no significant differences in biomass occurred between treatments or seasons. The lack of differences in biomass may indicate that colonisation space was at a premium with different, yet similar-sized epibiotic species occupying any available space. Despite the morphological differences between *Z*. *marina* and *S*. *muticum*, mobile invertebrates generally respond to macrophyte structure rather than being species-specific in choosing a host [3,55,60,61].

Differences between the life cycles of *Z*. *marina* which is perennial in this habitat and the annual *S*. *muticum* may also adversely affect epibiotic assemblages. Loss and fragmentation of *Z*. *marina* beds, whether through *S*. *muticum* competition or other anthropogenic disturbances, may be detrimental to the native epibiotic assemblages due to edge effects [44] and dispersal limitation at a variety of scales. Although *S*. *muticum* with its larger size and numerous branchlets can create an increase in habitat availability and structure [55], any habitat enhancements are ephemeral due to the annual senescence of its vegetative thallus [53,62,63]. If *S*. *muticum* replaces *Z*. *marina* and becomes the dominant macrophyte within the estuary, maintenance of the epibiota assemblage may not be possible even with the provision of the increased complexity and heterogeneity of *S*. *muticum*.

Loss of habitat may consequently reduce the overall productivity of a system. Epiphytes can play a critical role in the mean annual net production within seagrass meadows, with some epiphyte productivity as high as 20% (i.e., 200 g C m^-2^ yr^-1^) as reported in Florida and about 25% of the annual production in *Thalassia testitudinum* Banks ex König, 1805 meadows off the North Carolina coast [64]. In the Gulf of Mexico, Moncreiff and Sullivan [23] found that the epiphytes on *Halodule wrightii* Ascherson, 1868, contributed a greater proportion of organic matter to higher trophic levels than the seagrass itself using stable isotope analysis. Mittermayr [24] also indicated similar findings from a *Z*. *marina* ecosystem in the Baltic Sea. Therefore, loss of critical epiphytic substrata such as *Z*. *marina* can greatly influence the overall mean net production within seagrass ecosystems and the herbivores and foragers that rely on the presence of *Z*. *marina*.

The second primary mechanism we propose to underlie the effect we encountered is changes in the production of allelopathic / defensive compounds such as polyphenols [65–67] as either a direct or indirect consequence of the presence of *S*. *muticum*. Most marine plants and algae produce chemicals that act as anti-foulant or anti-herbivore agents [68]. Phenolics are amongst the most ubiquitous and their anti-herbivory activity is thought to derive primarily from their unpalatability or unacceptability to grazers [40,69,70]. For example, feeding experiments carried out in Portugal indicated that macro- and mesoherbivores preferred native red seaweeds over the introduced *S*. *muticum* potentially due to the high phenolic content found in phaeophytes [71]. Results from colorimetric assays conducted as part of this field study established that the phenolic content (both caffeic and tannic acid equivalents) of the leaves of *Z*. *marina* was significantly lower in the presence of *S*. *muticum* [52]. The lower phenolic content of the *Z*. *marina* shoots in the *Sargassum* treatment was concurrent with lower epibiota abundances, so it is likely that deterrence of grazers or sessile epibiota by polyphenolics was not the mechanism driving the assemblage differences found between treatments [72]. However, deterrence of grazers could result in generation of enemy-free space for many smaller epibiota and early stages of most sessile forms with lower rates of epibiota removal by indiscriminate surface grazers allowing epibiota to proliferate.

Whilst the majority of studies recount deleterious effects of *S*. *muticum* invasion, it is important to consider that not all marine invasions are necessarily detrimental. For example, Polte and Buschbaum [73] found that native pipefish in the Wadden Sea are promoted by the presence of *S*. *muticum*. Benthic organisms may also reap the benefits of invasive species presence as suggested by Vázquez-Luis et al. [14] who found that detritus from the invasive *C*. *racemosa* facilitated changes in species assemblages due to its ability to persist year round in the Mediterranean Sea. This benefit may not hold true in the case of *S*. *muticum*, however, as its biomass appears to simply disappear off-shore due to its annual senescence and degradation in late summer/early autumn.

## Conclusions

We developed a framework to detect changes in the epibiotic assemblage of *Z*. *marina* in the presence or absence of *S*. *muticum* using *in situ* manipulation. Results indicate that the overall epibiota biomass present on the blades is not significantly affected by manipulated density of *S*. *muticum* employed (4 plants m^-2^) despite significant differences in epibiota assemblages between the *Sargassum* and Control treatments. Although the presence of *S*. *muticum* may increase the overall habitat and spatial heterogeneity within the estuary, these benefits are restricted to seven or eight months a year due to the annual nature of the growth cycle of the invasive alga. Overall, the changes we recorded were subtle, highlighting the importance of longer-term studies in invasion biology. It is as yet unknown what, if any, cascading effects *S*. *muticum* invasion of seagrass beds in N. Europe will have, but indirect effects mediated by epibiota must be considered as well as direct effects. As the invasion process proceeds and invasive taxa become more prevalent, it seems unlikely that these consequences will not contribute to the general impoverishment of the environment and to the biotic homogenisation that often characterizes invaded systems [74].
